# Supplementary material for: Different Outcomes of Chicken Infection with UK-Origin H5N1-2020 and H5N8-2020 High-Pathogenicity Avian Influenza Viruses (Clade 2.3.4.4b)
Source: Viruses. 2023 Sep 12;15(9):1909. doi: 10.3390/v15091909 (PMC10537040; doi:10.3390/v15091909)
Supplement: Supplementary file 1 [file viruses-15-01909-s001.zip › viruses-2537651-supplementary.pdf]

(a) Opportunistic deaths following infection with H5N8-2020 (high dose):

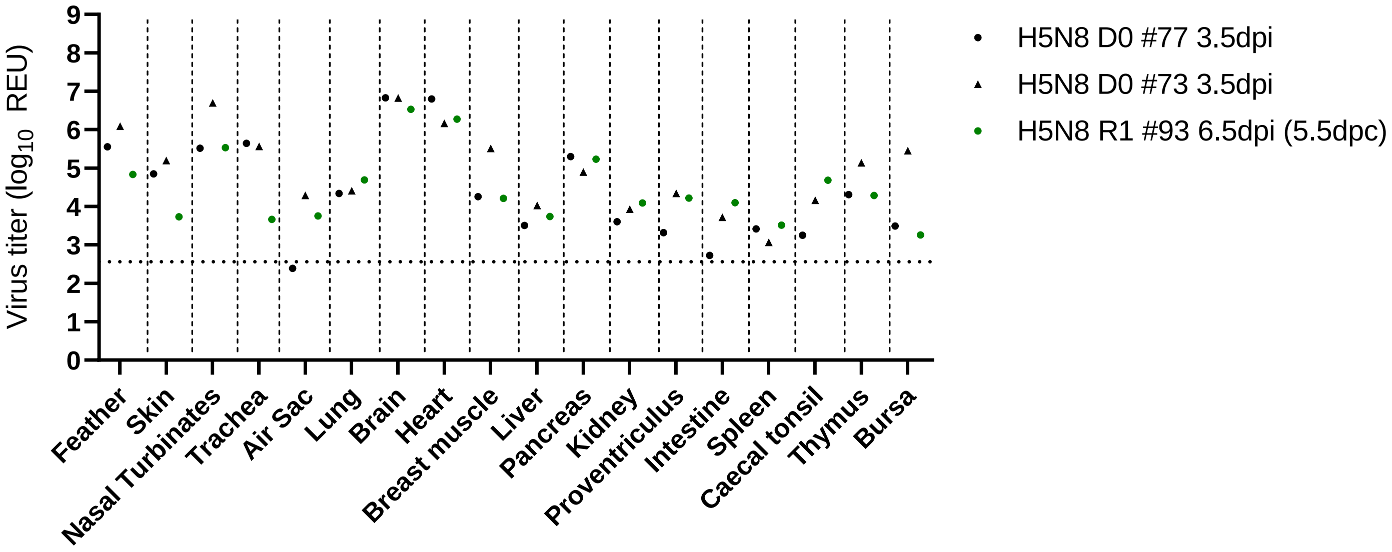

(b) Opportunistic deaths following infection with H5N8-2020 (medium dose):

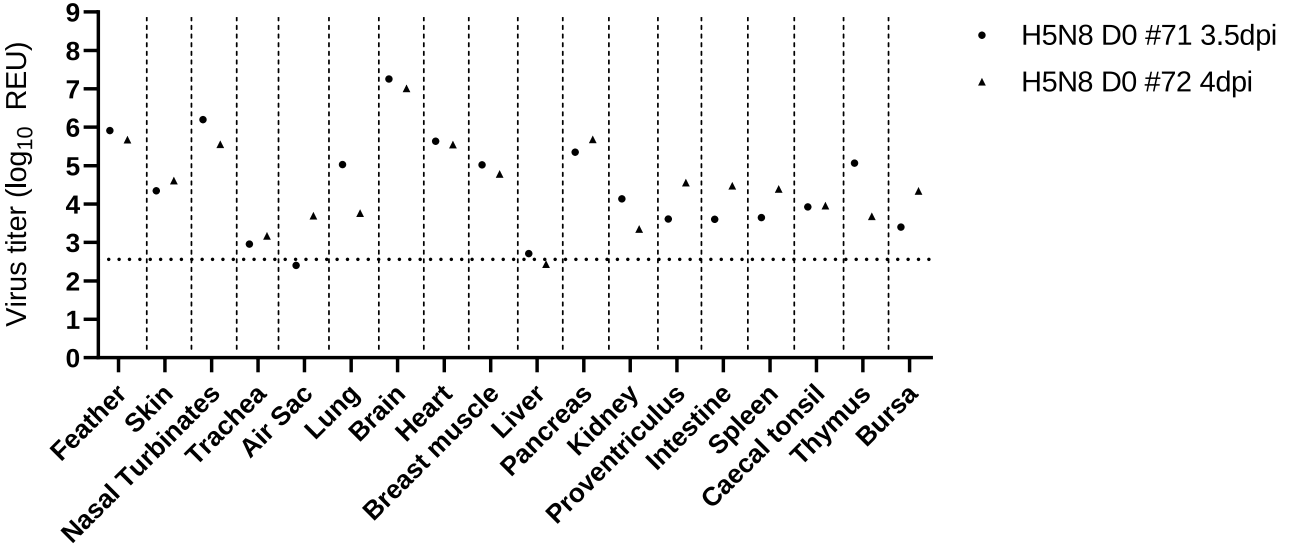

Figure S1. Viral RNA (vRNA) levels in chicken tissues obtained from opportunistic deaths during the H5N8-2020 MID_{50}/transmission experiment. Tissues were collected from (a) two chickens directly infected with the H5N8-2020 high dose plus the cohoused infected R1 contact; and (b) two chickens directly infected with the H5N8-2020 medium dose. Individual chicken ID #s and mortality times are indicated, see also Figure S3.

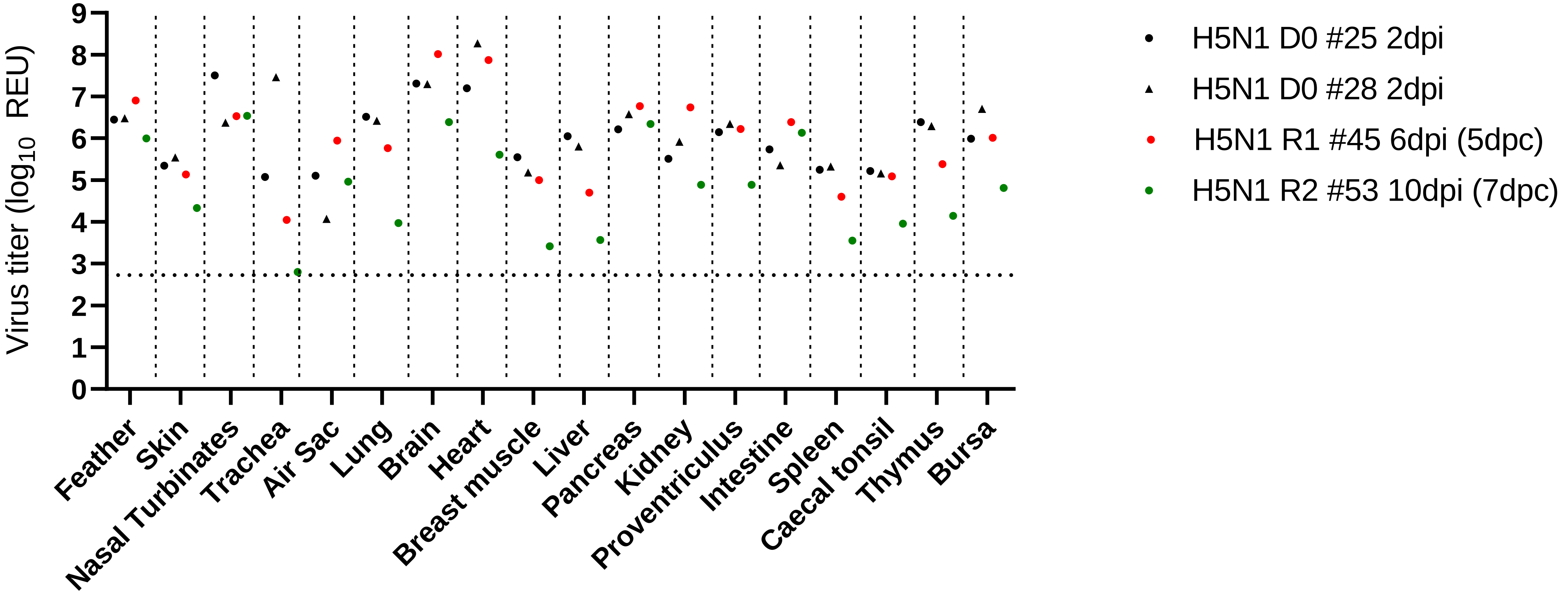

Figure S2. Viral RNA (vRNA) levels in chicken tissues obtained from opportunistic deaths during the H5N1-2020 MID_{50}/transmission experiment. Tissues were collected from two chickens directly infected with the H5N8-2020 high dose plus the cohoused infected R1 and R2 contacts. Individual chicken ID #s and mortality times are indicated, see also Figure S3.

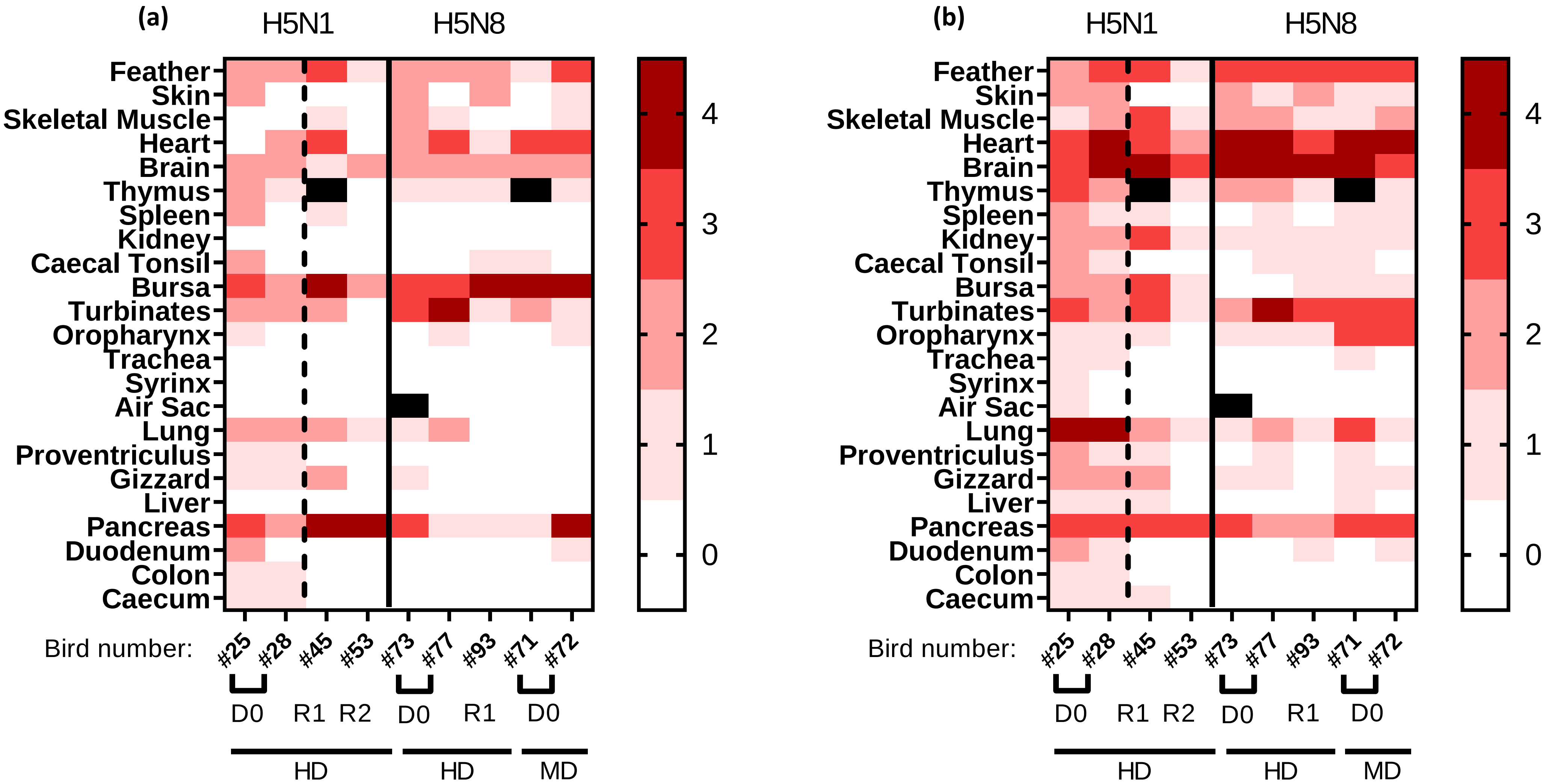

Figure S3. Summary of (a) histopathological changes and (b) virus-specific IHC staining observed in chicken tissues obtained from opportunistic deaths during the H5N8-2020 and H5N1-2020 MID_{50}/transmission experiment. For the scale of semi-quantitative scoring, please refer to Figure 4 legend. D0, directly infected; R1, first contact; R2, second contact; HD, high dose; MD, medium dose. Black squares indicate that no scoring was carried-out. Individual chicken ID #s are indicated, see also Figures S1 and S2 for the mortality times.
